# Global exposure to flooding from the new CMIP6 climate model projections

**DOI:** 10.1038/s41598-021-83279-w

**Published:** 2021-02-12

**Authors:** Yukiko Hirabayashi, Masahiro Tanoue, Orie Sasaki, Xudong Zhou, Dai Yamazaki

**Affiliations:** 1 Department of Civil Engineering, Shibaura Institute of Technology, 3-7-5 Toyosu, Koto-ku, Tokyo, 135-8548 Japan; 2 Center for Global Environmental Research, National Institute for Environmental Studies, 16-2, Onogawa, Tsukuba, Ibaraki 305-8506 Japan; 3 Graduate School of Environment Studies, Nagoya University, Furo-cho, Chikusa-ku, Nagoya, 464-8601 Japan; 4 Institute of Industrial Science, The University of Tokyo, 4-6-1, Komaba, Meguro-ku, Tokyo, 153-8505 Japan

**Keywords:** Civil engineering, Hydrology

## Abstract

Estimates of future flood risk rely on projections from climate models. The relatively few climate models used to analyze future flood risk cannot easily quantify of their associated uncertainties. In this study, we demonstrated that the projected fluvial flood changes estimated by a new generation of climate models, the collectively known as Coupled Model Intercomparison Project Phase 6 (CMIP6), are similar to those estimated by CMIP5. The spatial patterns of the multi-model median signs of change (+ or −) were also very consistent, implying greater confidence in the projections. The model spread changed little over the course of model development, suggesting irreducibility of the model spread due to internal climate variability, and the consistent projections of models from the same institute suggest the potential to reduce uncertainties caused by model differences. Potential global exposure to flooding is projected to be proportional to the degree of warming, and a greater threat is anticipated as populations increase, demonstrating the need for immediate decisions.

## Introduction

Flood risk is changing drastically worldwide, associated with socioeconomic growth and climate change. Global flood risk assessments have investigated the populations and assets potentially exposed to future flooding^1–7^, based on the multiple atmosphere–ocean general circulation model (AOGCM) in the Coupled Model Intercomparison Project Phase 5 (CMIP5)^8^. Some studies have focused on human exposure at different levels of warming^2,3,9^, which makes an important scientific contribution for implementing adequate mitigation and adaptation targets. However, due to limitations in the available daily runoff data in AOGCM, few studies have focused on the uncertainties of these projections^1^. In this study, we made the first comparison of flood projections from CMIP5 and CMIP6 and investigated the model uncertainties using multi-model ensembles.

**Figure 1:**
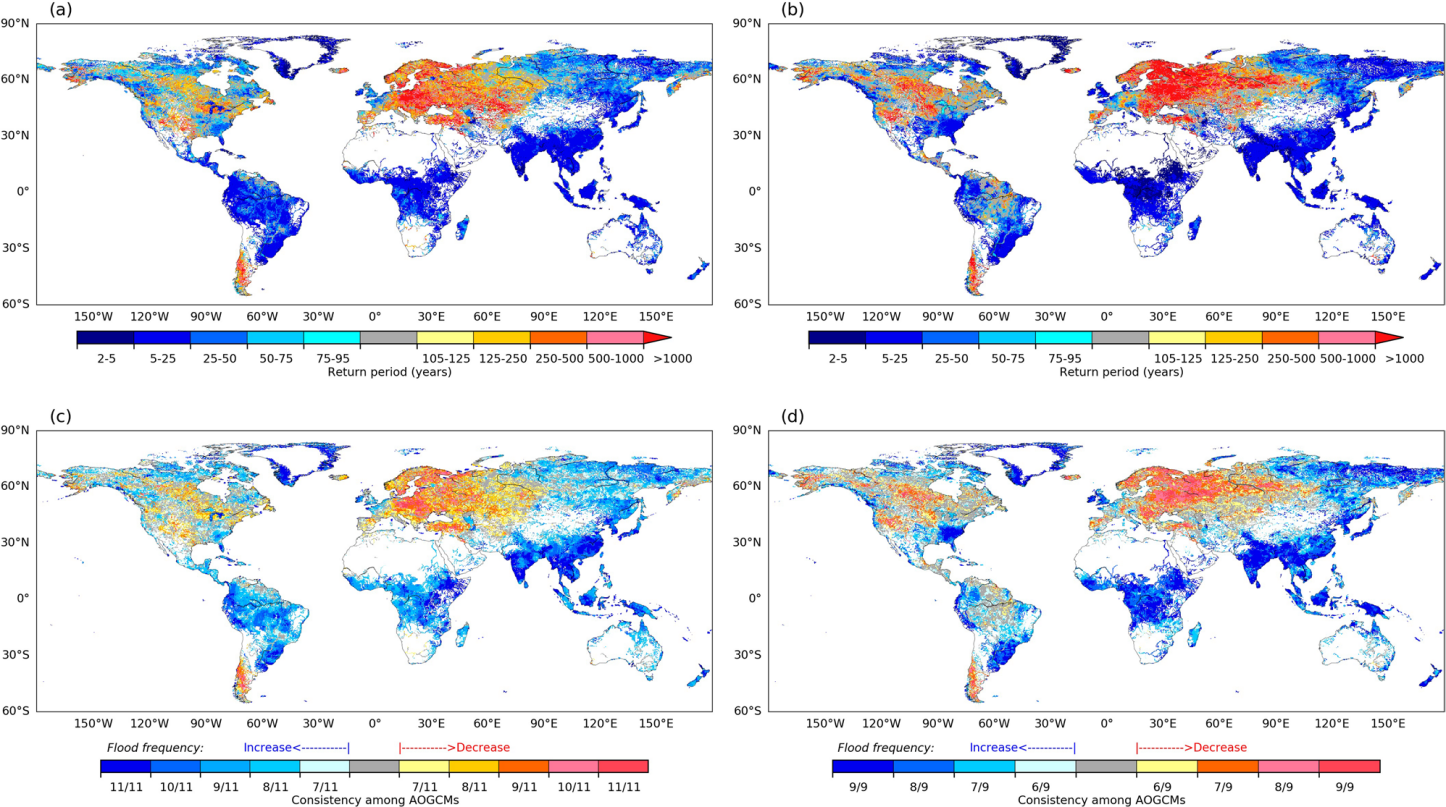
Projected change in flood frequency. Multi-model median return period (years) in future (2071–2100) for discharge corresponding to a 100-year flood in the past (1971–2000), and for (**a**) CMIP5 and (b) CMIP6 under the RCP8.5 and ssp585 (SSP5-RCP8.5) scenarios, respectively, and model consistency for (**c**) CMIP5 and (d) CMIP6. Grid cells with a mean annual discharge of a retrospective simulation for 1971–2000 of < 0.05 mm^−1^ day are screened out (see “Methods”). This figure was created using python 2.7.12.

Figure 1 shows the change in flooding between the ends of the last (1971–2000) and current (2071–2100) centuries according to the CMIP5 and CMIP6 models under the highest emission scenario, Representative Concentration Pathway 8.5 (RCP8.5). Following previous studies^1^, we expressed the change in flooding as the change in the return period (probability) of a river discharge having a 100-year return period in the past. The time series of simulated past and future annual maximum daily river discharge were fitted to an extreme distribution function and the multi-model median of the future return period of river discharge and agreement among multi-model ensembles were calculated for each grid cell. Further details of the processes and modelling framework are provided in the “Methods” section.

The overall patterns of increase and decrease in flood frequency (corresponding to decreases and increases in the return period, respectively) are remarkably similar with CMIP5 and CMIP6, with increases in many regions in South Asia, Southeast Asia, Northeast Eurasia, eastern and low-latitude Africa, and South America and decreases in northern and eastern Europe, Anatolia, Central Asia, central North America, and southern South America. The results of three other RCP scenarios (Supplementary Fig. S2) showed similar spatial distributions. The result indicates that the large-scale features of flood projection are robust to the resolution and assumptions of the models, despite the substantial development of climate models since CMIP5. Differences in the direction of change in Texas (USA), the Amazon, Italy, and South Africa were not caused by differences among the models used. Our comparison of return period change data obtained by six models from institutions that participated in both CMIP5 and CMIP6 showed very similar differences in the spatial patterns of flood frequency changes (Supplementary Fig. S3). Model consistency was low for Texas, Italy, and South Africa in CMIP5, and in the Amazon in CMIP6, which was the main reason for differences in the direction of changes between CMIP5 and CMIP6.

The consistency of the future direction of flood change between CMIP5 and CMIP6 was also very similar in many regions where flood frequency increases (*e.g*., Asia, Northeast Eurasia, low-latitude Africa, and South America) or decreases (Scandinavia and central to eastern Europe and high-latitude South America). Of the global model grid cells, 36% showed increased flood frequency with relatively high consistency (more than 7 of the 7 AOGCMs). By contrast, 15% of the global grid cells had a relatively high consistency (7 of 9) among the AOGCMs in the regions where the flood frequency decreases in the future.

**Figure 2:**
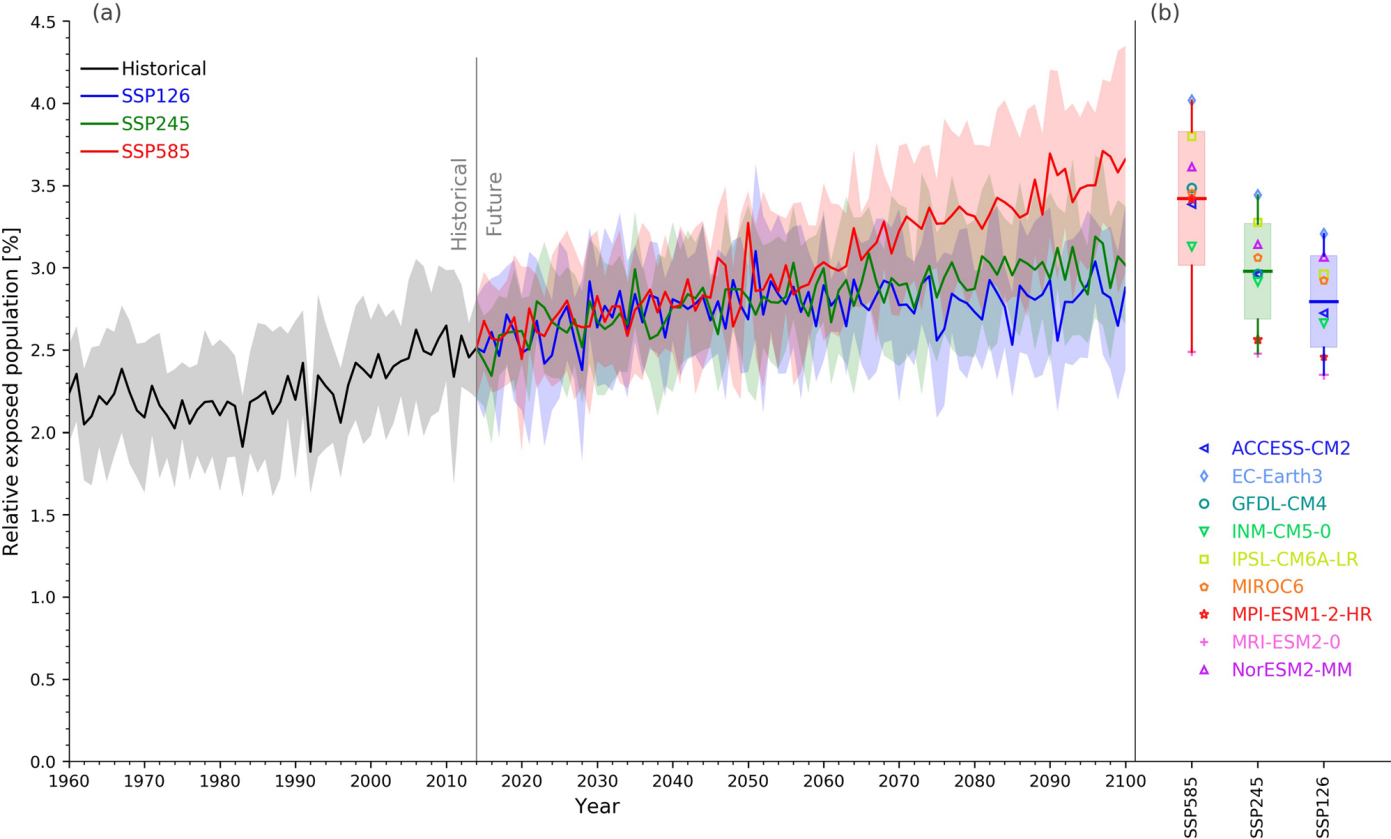
Global potential flood exposure change and uncertainty. Global potential flood exposure change (mean and one standard deviation among AOGCMs, indicated by as shading) for the multi-model median return period (years) in the future (2071–2100) for a discharge corresponding to a 100-year flood in the past (1971–2000).

Figure 2 shows the multi-model mean population potentially exposed to large amounts of flooding from the middle of the twentieth century to the end of the twenty-first century. There is high inter-annual fluctuation due to the relatively small number of ensembles and the fact that the exposure is largely affected by rare large floods. A difference in the potential flood exposure becomes obvious after around 2070.

**Figure 3:**
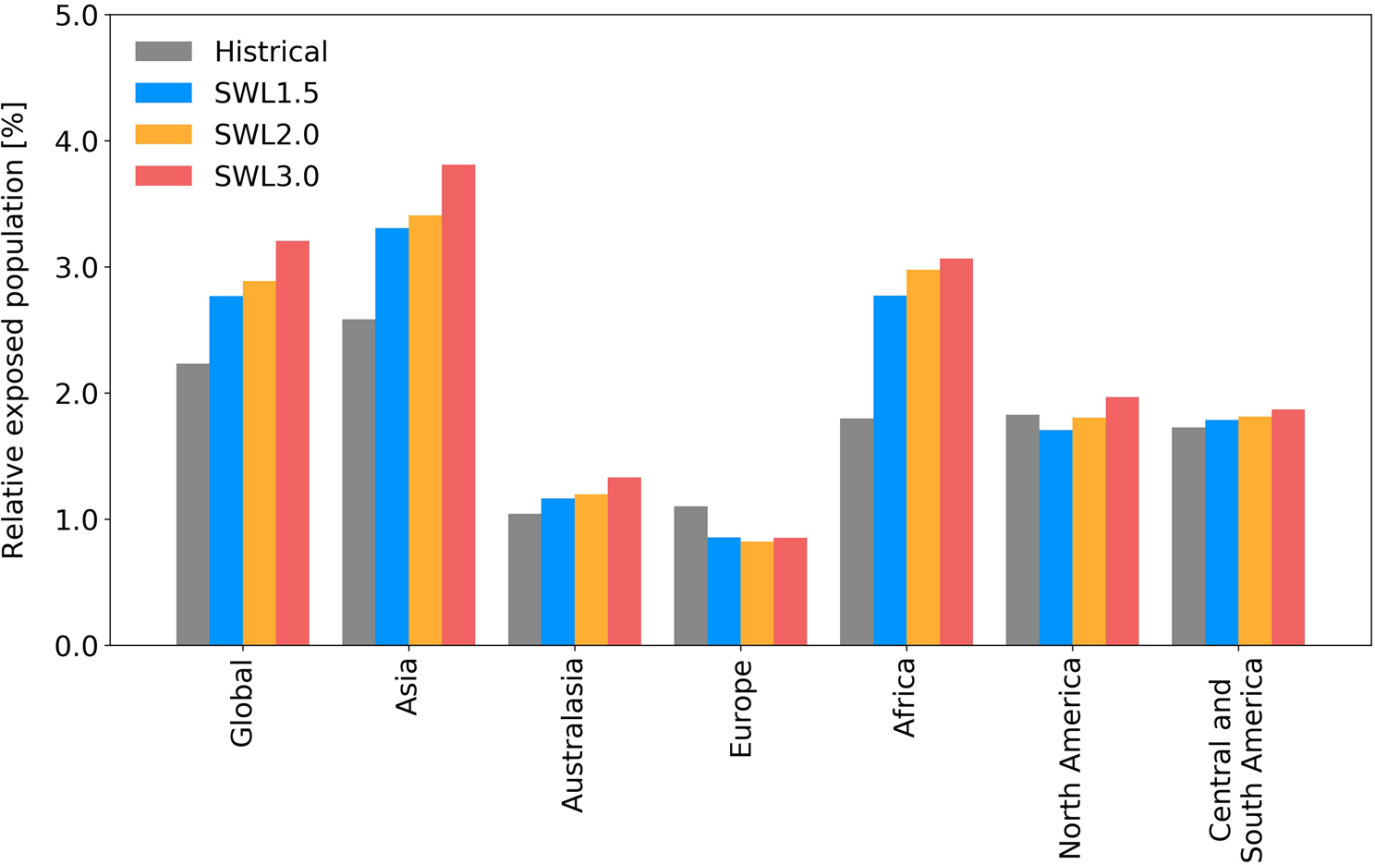
Potential flood exposure during the baseline period and projected future warming levels. The ssp585 scenario (SSP5 and RCP8.5) was applied to different regions. The percentage of the regional total population (fixed at the 2015 level) is shown for each region.

Following previous research^10^, we compare the robustness of global flood exposure change to the specific warming level (SWL). The range of exposure for the twenty-first century is not simple to compare, because CMIP5 analyzed 11 AOGCMs while CMIP6 analyzed 9 AOGCMs due to data availability. Moreover, the experiments selected AOGCMs from different institutes (Tables S1 and S2). However, the increase in potential flood exposure associated with SWLs (1.5 °C, 2 °C and 3 °C warming) is very similar with CMIP5 and CMIP6 (Fig. 3). To focus on flood change only, the population distribution was fixed at that of 2015; the result shows that annual global flood exposure increases about 1.4-fold (from 2.2 to 3.2% of the global population) from historical period (1971–2000) with 3 °C warming in CMIP6.

The potential flood exposure varies among regions depending on the population in flood-prone regions and the projected flood change (Fig. 3). Increased potential flood exposure due to warming was observed in Asia, North America, and Africa, whereas exposure was stable or lower at higher SWLs in Europe and North Africa. The increase in flood exposure was high in Africa and Asia, particularly with 3 °C warming (1.7-fold and 1.5-fold higher than the average of 1971–2000, respectively), reflecting a significant increase in flooding (Fig. 1b).

We analyzed the potential number of people, to focus only on the changes associated with climate change. Hence, the value is larger than the estimates of previous studies analyzing large floods^1^ or those considering local flood protection. The increase in global potential flood exposure is due mainly to increased exposure in regions such as Asia and Africa, where flood frequency is projected to increase (Fig. 1). The model spread relative to the model mean change is also very similar, implying that the models’ projections have not converged.

**Figure 4:**
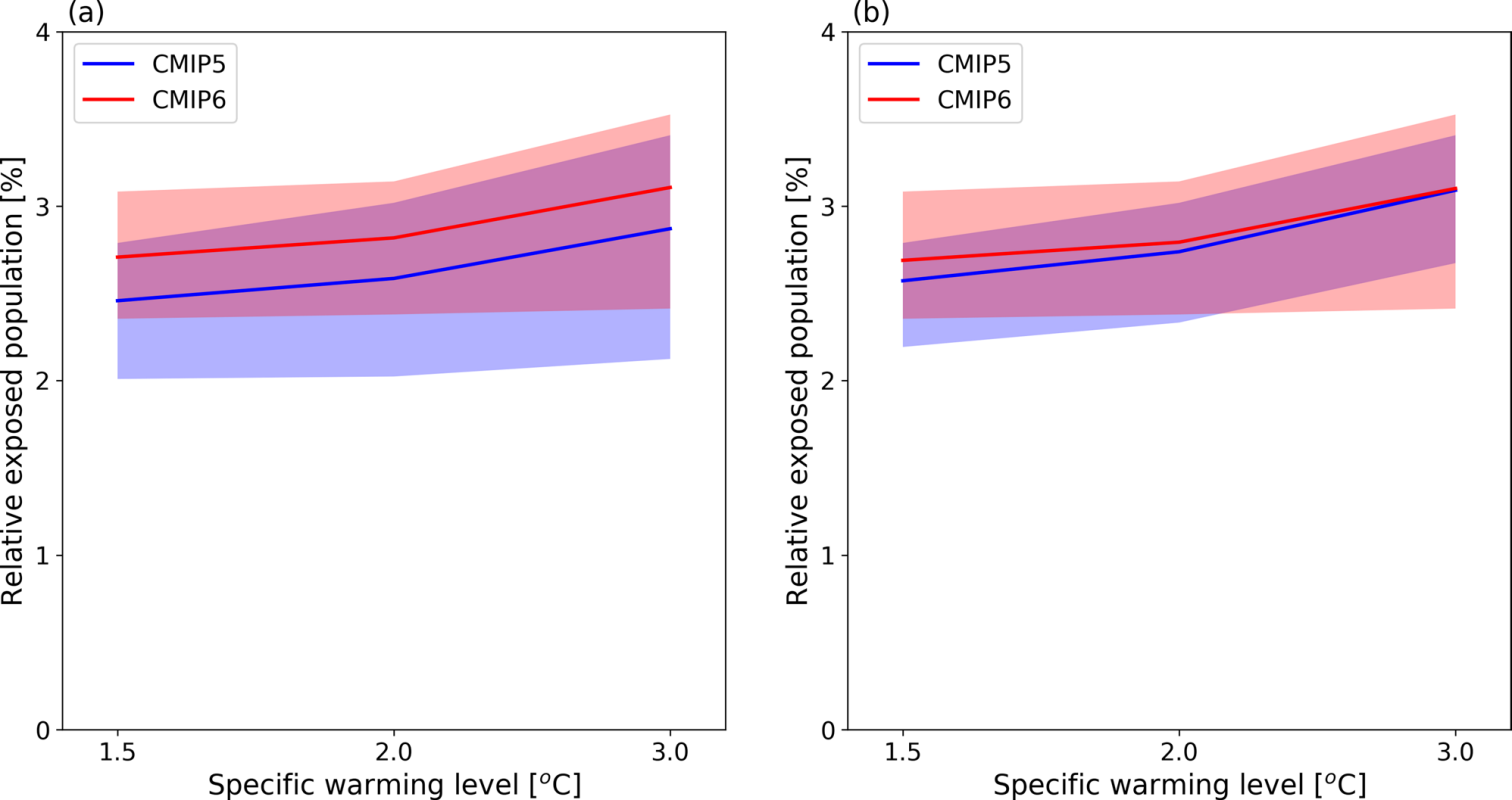
Model robustness for flood exposure. (**a**) Fraction of the global population exposed to potential floods using CMIP5 and CMIP6 corresponding to a SWL relative to the preindustrial period. The mean and maximum and minimum ranges among AOGCMs (lines and shaded area, respectively) are shown. (**b**) As (**a**), but for a subset of 6 models from 6 institutions participating in both CMIP5 and CMIP6 (Supplementary Tables S1 and S2). The population distribution is fixed at the 2015 level.

The assumption that AOGCMs from the same institute show similar results was confirmed in for flood exposure of a selected subset of AOGCMs used in both CMIP5 and CMIP6 (Fig. 4b, Tables S5 and S6). This finding indicates that although model spread due to internal climate variability is irreducible, the uncertainty arising from model differences is large and can potentially be reduced.

We examined potential flood exposure focusing only on changes induced by climate change; however, flood risk also depends on other drivers of change, such as the degree of socioeconomic development and associated vulnerability and exposure^11,12^. For example, previous studies have demonstrated that vulnerability to flooding has changed over time in association with economic development^11,12^, which affected flood risk projections^6,12^. A newly developed global data of flood protection standard allowed us to estimate the consequences of a given flood exceeding the current flood protection level^13^. Studies have shown that reducing flooding vulnerability greatly reduces the future projected consequences of flooding^2–4,12^. Moreover, increases in assets and population in a flood-prone region can increase flood exposure. Thus, flood exposure projections were underestimated in cases where the population will increase in the future. Despite the model development including finer resolutions of topography and population distribution, potential flood exposure for large floods was similar to those projected in a previous study^1^.

Projection uncertainty arises in association with multiple models. We analyzed uncertainty using a bootstrap method; future changes in potential flood exposure were evaluated using non-parametric bootstrap samples^14^. A random number generator was used to sample a 109-year subset of exposure and surface temperature anomaly from the 109-year samples (30-year moving average for 1960–2100). This procedure was repeated 1000 times, giving 1000 exposure and temperature anomaly estimates for each AOGCM. The 5th and 95th percentiles, and average, of the 1000 estimates of potential flood exposure were then obtained for both CMIP5 and CMIP6; the ranges were similar between CMIP5 and CMIP6.

In summary, it is clear that potential flood exposure will increase with the level of warming, even for a fixed population. Moreover, the value will increase with increasing population, particularly at lower latitudes. Despite the uncertainty remaining in the global data and modeling framework, the robustness of the flood projection presented here supports the efforts to make decisions needed to adapt to climate impacts and mitigate greenhouse gas emissions.

## Methods: river discharge simulation

We calculated the discharge from AOGCMs participating in CMIP5^8^ and CMIP6^15^ (listed in Supplementary Tables S1 and S2). The AOGCMs were selected from independent institutions to avoid the potential dependence of different versions of the AOGCMs from the same institution. River discharge is calculated along the river network through a high-resolution (15′ × 15′ spatial resolution) global river network map using the Catchment-based Macro-scale Floodplain Model (CaMa-Flood v4.0)^16^ and daily AOGCM runoff data. CaMa-Flood simulates river water levels and floodplain inundation hydrodynamics based on high-resolution (~ 500 m) sub-catchments and reasonably represents the temporal variation and peaks of river discharge (Fig. S4). The inundation area showed reasonable correspondence in lowland areas due to the river bifurcation scheme of the model^17,18^.

When several ensemble runs were available, the first ensemble of each AOGCM was selected and disaggregated into 0.5° pixels through bi-linear interpolation. Validation of the modeled historical discharge against in situ observation from the Global Runoff Data Centre showed reasonable consistency between simulation- and observation-based annual discharges and annual maximum daily discharges (the selected river basins and details of the validation are presented in Supplementary Information S2). Noted that CaMa-Flood does not consider the effects of anthropogenic river management, including the regulation of floodwater.

## Methods: fitting an extreme distribution function

The annual maximum daily discharge was fitted to the two-parameter Gumbel distribution^19^, with parameters estimated using the probability-weighted moments method^20^. We used 30-year periods to represent past and future floods. Due to the relatively small data samples, we used the Gumbel distribution because it provides relatively robust and stable results from small data samples compared to other distributions^1,21^. A previous study showed that randomly increased samples produced similar flood change signals and model consistency^1^. The Gumbel distribution can yield higher probabilities of extreme values than other distributions because it has lighter tails^3^; however, frequency changes can be well illustrated. We evaluated the goodness of fit of the simulation data to the Gumbel distribution based on the probability plot correlation coefficient (PPCC)^22^, which has been widely used in hydrology to evaluate agreement between estimated distributions and the original data. For all AOGCMs, ~ 79 ± 7% of the global model grid cells over land, excluding dry regions (average of the modeled 30-year (1971–2000) mean annual discharge at < 0.05 mm day^−1^), had a PPCC > 0.96 (significant at the 95% level; Fig. S1). Due to the small inundation extent, grids poor fit to the Gumbel distribution did not affect the flood exposure calculation.

The magnitude of river discharge corresponding to the 100-year return period in the past (1971–2000) was first computed using the annual maximum daily discharge of the historical AOGCM simulation fitted to the Gumbel distribution. The return period of this calculated discharge in the future (2071–2100) was then computed for each AOGCM. The median return period of the 9 (CMIP6) or 11 (CMIP5) AOGCMs was then obtained. Finally, the consistency among the AOGCMs was calculated by counting the number of AOGCMs showing the same sign of change (increase or decrease in frequency).

## Methods: exposure calculation

The population potentially exposed to flooding was calculated as the sum of the population over the inundated area modeled. The modeled inundation area was overlaid onto the population dataset of version 4 of the Gridded Population of the World (GPWv4)^23^. To focus on climate change only, a fixed population distribution corresponding to that of 2015 was used.

Because we used the direct output of runoff from the AOGCMs without correcting the bias related to the lack of gauge observations on a global scale, we did not calculate inundation areas and associated flood exposure directly from discharge values. Instead, we followed a published approach^1,2^, and linked the recurrence frequency (return period) in each 0.25° pixel (15 arcmin, ~ 25 km at the equator) to water depth at a finer spatial resolution (30 arcsec resolution, approximately 1 km at the equator) according to the relationship between frequency (return period of annual maximum river water storage) and inundation area. This was based on a retrospective model simulation forced by observation-based climate data (i.e., a retrospective simulation). For the retrospective simulation, water depth in each 0.25° pixel (i.e., water level above the top of a river channel) was downscaled using a 30 arcsec (~ 1 km at the equator) high-resolution digital elevation model (DEM)^18^, by comparing the elevation of pixels in the DEM with the water level modeled at a coarse resolution. Since CaMa-Flood uses the same high-resolution sub-grid topography for both river routing and downscaling, the water volume is consistent at 0.25° pixel resolution before and after downscaling. Finally, a look-up table of the return period at a 0.25° pixel resolution (2-year to 1,000-year) and inundation area at 30 arcsec was used to calculate flood exposure for each AOGCM.

The annual maximum daily discharge of the retrospective simulation from 1971 to 2000 was first fitted to a generalized extreme value distribution. Then, the discharge magnitude corresponding to the return periods from 2 to 1000 years was calculated for each grid cell. Simultaneously, the water level was downscaled using a bias-corrected high-resolution DEM, the Multi-Error-Removed Improved-Terrain (MERIT) DEM^24^ to obtain a finer inundation area to obtain look-up tables for each return period (2–1000 years) at 0.25° pixel resolution and the corresponding inundation extent at 30 arcsec resolution.

For each AOGCM simulation, the annual maximum daily discharge of the historical simulation from 1971 to 2000 was fitted to a generalized extreme value distribution in each 0.25° pixel and distribution parameters were calculated (see “Methods: fitting an extreme distribution function”). Using the extreme parameters, the return period of the annual maximum daily discharge at each year and corresponding inundation extent at 30 arcsec resolution were calculated for 1971–2100. Population data at 30 arcsec resolution was overlaid onto the 30 arcsec inundation area.

We present results at each SWL of 1.5 °C, 2 °C and 3 °C above the preindustrial temperature (Supplementary Tables S3 and S4). Following previous research^2^, SWLs were calculated as the year each SWL was first passed from a reference year in the preindustrial period (1850–1900), using a running mean of the 30-year global averaged annual mean temperature.

## Supplementary Information

Supplementary Information.
