# Supplementary material for: Global exposure to flooding from the new CMIP6 climate model projections
Source: Sci Rep. 2021 Feb 12;11:3740. doi: 10.1038/s41598-021-83279-w (PMC7881105; doi:10.1038/s41598-021-83279-w)
Supplement: Supplementary file 1 — Supplementary Information. [file 41598_2021_83279_MOESM1_ESM.pdf]

Global exposure to flooding from the new CMIP6 climate model projections

Supplementary Information

Yukiko HIRABAYASHI*[1], Masahiro TANOUE[2], Orie SASAKI[3], Xudong ZHOU[4] and Dai YAMAZAKI[4]

[1]Department of Civil Engineering, Shibaura Institute of Technology, Japan

[2]Center for Global Environmental Research, National Institute for Environmental Studies, Japan

[3]Graduate School of Environmental Studies, Nagoya University, Japan

[4]Institute of Industrial Science, The University of Tokyo, Japan

Summary

S1. Graphs and tables illustrating the Methods

S2. Additional results

S3. Model evaluation

S4. Discussion of uncertainty

S1. Graphs and Tables Illustrating the Methods

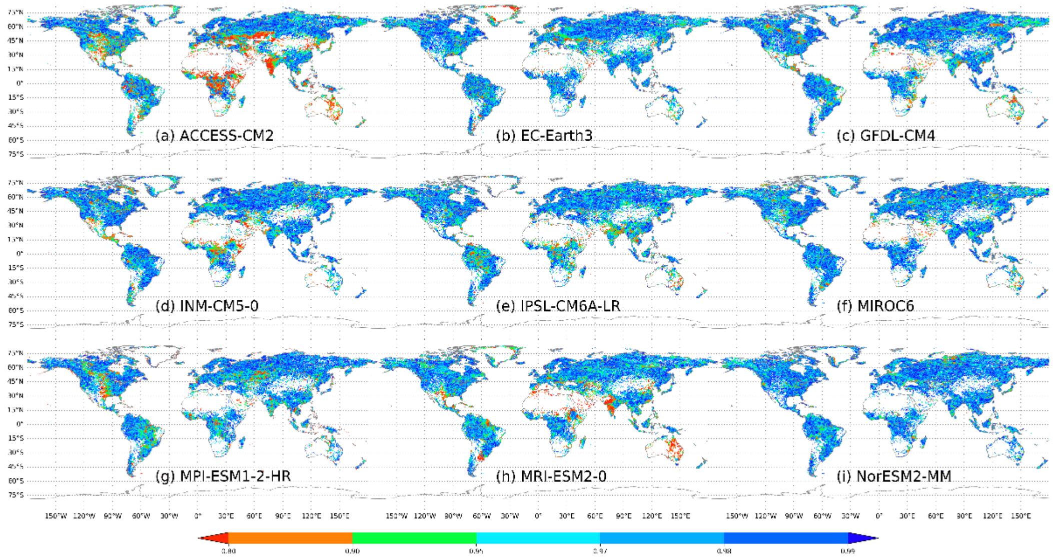

Figure S1. Probability plot correlation coefficient test for historical (1971-2000) AOGCM simulations. This figure was created using python 2.7.12.

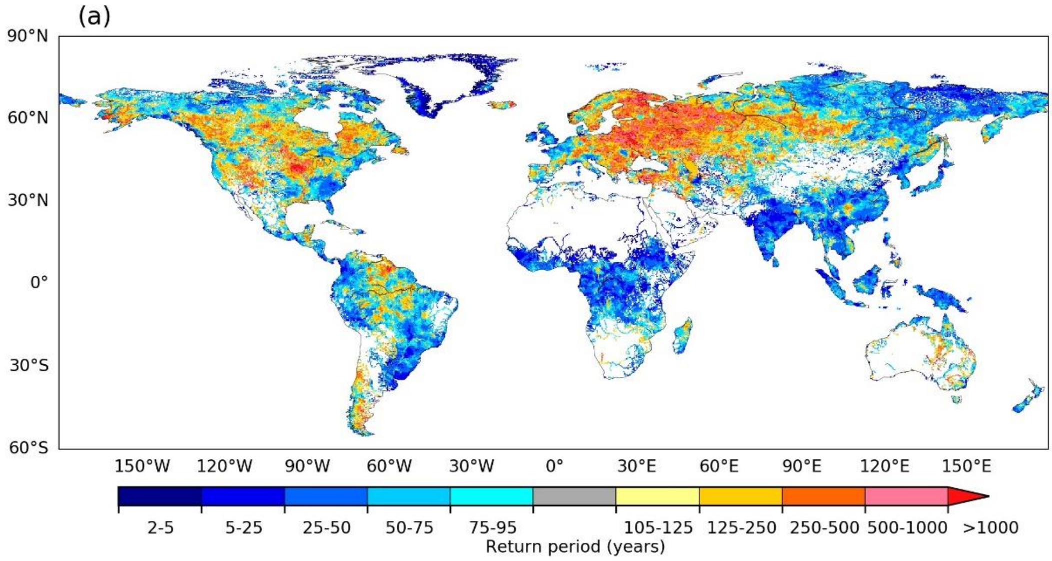

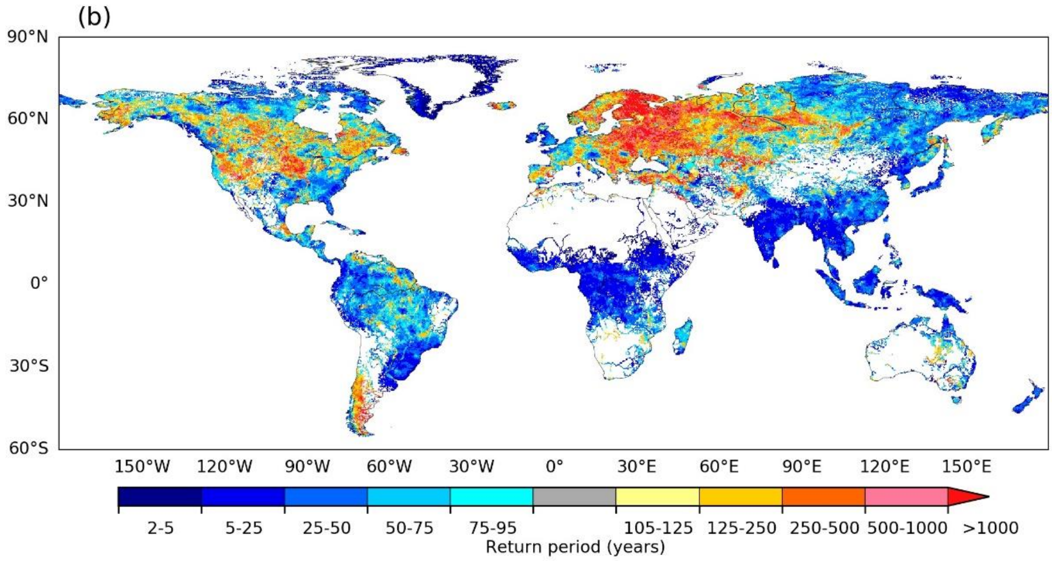

Figure S2. The same for Fig.1, but for (a) RCP2.6 and ssp126 (SSP1 and RCP2.6), and (b) RCP4.5 and ssp245 (SSP2 and RCP4.5). This figure was created using python 2.7.12.

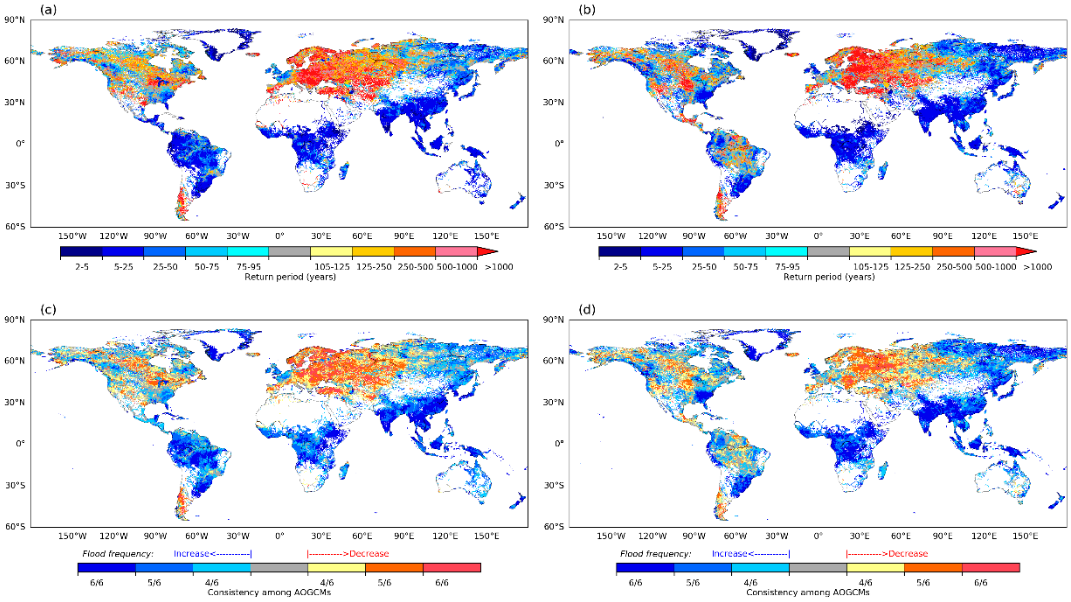

Figure S3. The same for Fig.1, but using GCMs that joined both CMIP5 and CMIP6. This figure was created using python 2.7.12.

Table S1. The AOGCMs analyzed in this study. The institution and model names were taken from the file name with available experiments. Asterisks indicate institutions analyzed in both Hirabayashi et al. (2013)[1] and this study.

Model	Institution	Scenario availability (number of models is given in brackets)		
		SSP1 RCP2.6 (ssp126) (9)	SSP2 RCP4.5 (ssp245) (8)	SSP5 RCP8.5 (ssp585) (10)
ACCESS-CM2	Commonwealth Scientific and Industrial Research Organization, Australia	x	x	x
EC-Earth3	ECMWF, EU	x	x	x
GFDL-CM4*	Geophysical Fluid Dynamics Laboratory, USA		x	x
INM-CM5-0*	Institute for Numerical Mathematics, Russia	x	x	x
IPSL-CM6A-LR	Pierre Simon Laplace Institute, France	x	x	x
MIROC6*	AORI, NIES, JAMSTEC, Japan	x	x	x
MPI-ESM-1-2-HR*	Max Planck Institute for Meteorology (MPI-M), Germany	x	x	x
MRI-ESM2-0*	Meteorological Research Institute, Japan	x	x	x
NorESM2-MM*	Norwegian Climate Centre, Norway	x	x	x

Table S2. The 11 AOGCMs in CMIP5 analyzed in Hirabayashi et al. (2013)[1].

Model	Institution
BCC-CSM1.1	Beijing Climate Center, China Meteorological Administration,China
CanESM2	Canadian Centre for Climate Modelling and Analysis, Canada
CMCC-CM	Centro Euro-Mediterraneo per I Cambiamenti Climatici,Italy
CNRM-CM5	Centre National de Recherches Meteorologiques/Centre Europeen de Recherche et Formation Avancees en Calcul Scientifique, France
CSIRO-Mk3.6.0	Commonwealth Scientific and Industrial Research Organisation in collaboration with the Queensland Climate Change Centre of Excellence, Australia
GFDL-ESM2G*	Geophysical Fluid Dynamics Laboratory, USA
INM-CM4*	Institute for Numerical Mathematics, Russia
MIROC5*	Atmosphere and Ocean Research Institute, National Institute for Environmental Studies, and Japan Agency for Marine-Earth Science and Technology, Japan
MPI-ESM-LR*	Max Planck Institute for Meteorology (MPI-M), Germany
MRI-CGCM3*	Meteorological Research Institute, Japan
NorESM1-M*	Norwegian Climate Centre, Norway

Table S3. The time to reach SWLs from the preindustrial global mean temperature (defined as 1850-1900) under the scenario of ssp585.

Model	Specific warming levels			
	1.5	2.0	3.0	4.0
ACCESS-CM2	2026	2038	2056	2071
EC-Earth3	2024	2036	2057	2074
GFDL-CM4	2030	2041	2061	2079
INM-CM5-0	2031	2046	2074	–
IPSL-CM6A-LR	2018	2033	2051	2066
MIROC6	2039	2053	2076	2095
MPI-ESM-1-2-HR	2033	2049	2074	–
MRI-ESM2-0	2026	2039	2064	2084
NorESM2-MM	2041	2054	2076	–

Table S4. The same as Table S2, but for the CMIP5 experiments analyzed in Hirabayashi et al (2013)[1] under the RCP8.5 scenario.

Model	Specific warming levels			
	1.5	2.0	3.0	4.0
bcc-csm1-1	2019	2036	2060	2083
CanESM2	2014	2027	2049	2069
CMCC-CM	2031	2043	2064	2081
CNRM-CM5	2033	2047	2069	–
CSIRO-Mk3-6-0	2034	2046	2066	2083
GFDL-ESM2G	2040	2057	2083	–
Inmcm4	2046	2060		–
MIROC5	2034	2049	2073	–
MPI-ESM-LR	2022	2040	2065	–
MRI-CGCM3	2040	2052	2075	
NorESM1-M	2035	2050	2075	

Table S5. Global potential flood exposure at SWLs (millions) under the scenario of ssp585 of CMIP6.

Model	Specific warming levels			
	1.5	2.0	3.0	4.0
ACCESS-CM2	184	193	212	233
EC-Earth3	221	229	255	273
GFDL-CM4	208	213	223	243
INM-CM5-0	199	207	221	–
IPSL-CM6A-LR	187	200	223	246
MIROC6	202	213	242	–
MPI-ESM-1-2-HR	185	199	238	–
MRI-ESM2-0	173	174	177	184
NorESM2-MM	226	230	258	–

Table S6. The same as Table S6, but for the CMIP5 experiments analyzed in Hirabayashi et al (2013)[1] under the RCP8.5 scenario.

Model	Specific warming levels			
	1.5	2.0	3.0	4.0
bcc-csm1-1	200	209	235	249
CanESM2	168	175	192	201
CMCC-CM	176	185	211	224
CNRM-CM5	179	184	198	–
CSIRO-Mk3-6-0	147	148	156	168
GFDL-ESM2G	167	179	206	–
Inmcm4	188	196	–	–
MIROC5	204	221	250	–
MPI-ESM-LR	188	198	211	–
MRI-CGCM3	161	171	196	
NorESM1-M	201	217	250	

S2. Model evaluation

Historical AOGCM simulation evaluated at 174 selected river basins using *in situ* observations obtained from the Global Runoff Data Center. River basins larger than 150,000 km^2 and with at least 30 years of data within the period 1970-2013 were selected. The average annual mean, annual maximum daily discharge, and discharge corresponding to the 100-year return period of the past (1971-2013) are evaluated in Figure S4. Among the 174 selected catchments, the average discharge compared well with observations, with a bias of <50% in 107 catchments. The mean annual maximum daily discharge had a slightly larger spread than the mean annual discharge, but was also within an acceptable range; 80 catchments had bias of < 50%. The overall bias for all basins for both the mean annual and mean annual maximum discharges was 7.0% and 24.3%, respectively. Large bias occurred for catchments with averaged observed discharge less than $1000m^3s^{-1}$. The proportion of gauges with less than 50% bias increased from 61.5% in the entire group to 83.1% for the group with large discharges. The proportion of bias less than 20% increased from 39.7% to 53.4%. The model also performs for the annual maximum discharge. The simulated multi-model median discharge corresponding to the 100-year return period was over-predicted compared to observations, with an overall bias of 30.6 %; 76 of 174 basins had a bias of < 50%.

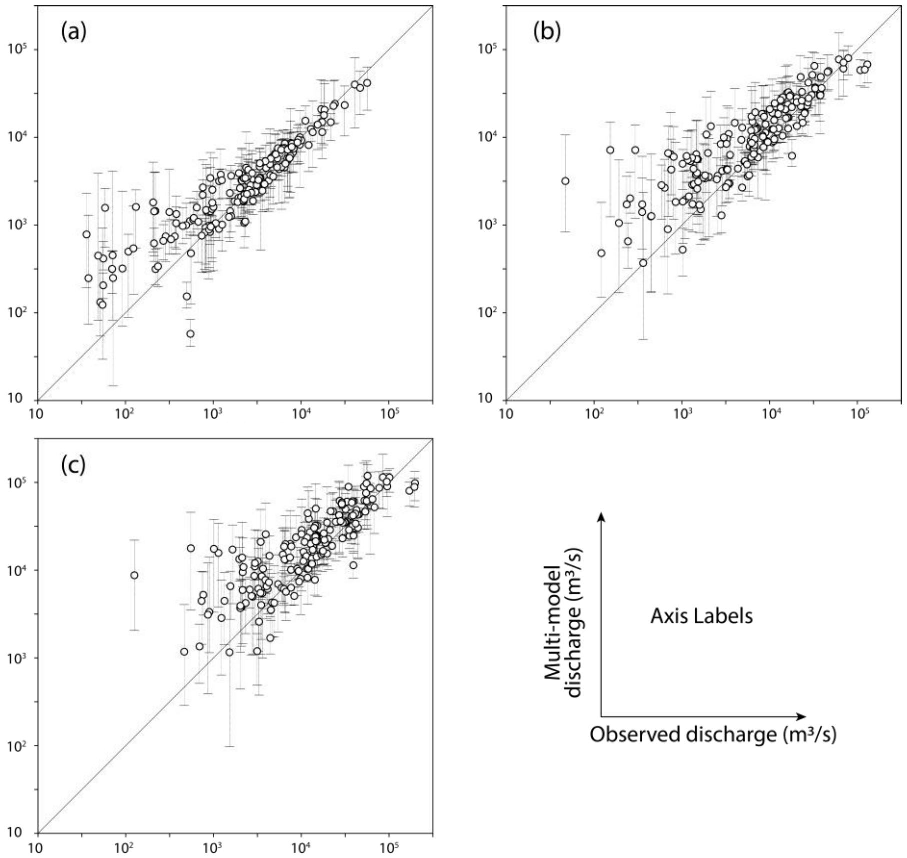

Figure S4. Comparison of the observed and simulated multi-model mean river discharges in past (1971–2000) for 174 selected river basins: (a) annual discharge, (b) annual maximum daily discharge, and (c) discharges with 100-year return periods. Error bars indicate the maximum and minimum values among for the 9 AOGCMs.
